# The Common Point of Countries Successful Policies in the Struggle Against COVID-19: Women Leaders

**DOI:** 10.1177/21582440231179458

**Published:** 2023-06-06

**Authors:** Süheyla Üçışık Erbilen, Merve Uysal

**Affiliations:** 1 Eastern Mediterranean University, Famagusta, Turkey; 2 Final International University, Kazafani, Cyprus

**Keywords:** COVID-19, women leaders, discourse analysis technique, leadership qualities, biopower

## Abstract

During the COVID-19 pandemic, which is one of the biggest epidemics of the last century and can be regarded as a global tragedy, leaders had to mobilize many resources of their countries quickly and persuade their citizens to change their routine behavior. The approach followed by the leaders of the country in their efforts to convince their people has been an important factor in their success or failure. This paper aims to examine with Michel Foucault’s notion of biopower, and discourses and behaviors of women leaders in countries against the global pandemic which cost high life tool gave harsh messages to the humanity. For this purpose, leadership examples in Finland, Iceland, Taiwan and New Zealand will be examined in detail using the discourse analysis technique. As a result, in current times when populist and autocratic leader style is on the rise, women leaders not only took their countries to success, but they also managed to inspire other countries. More importantly, the struggle of women leaders against the pandemic revealed that a different management style is possible.

## Introduction

People starting to live collectively brought about the need for management. Forms of management, which come from the past to the present following a changing course, is in constant motion by focusing on the human beings depending on globalization, revolutions, wars, organizational structures, and developments in information and communication technologies. Individuals continue to produce new management theories with the purpose of keeping up with developments. Considering classical theories of power, they are approaches which emphasize economic and legal models. According to the economic power model underlined by Marxism, power is based on class domination and acting in line with economic interests. Legal power model, on the other hand, analyses power within the framework of law, ethical rights and political power. The French philosopher Michel Foucault, the representative of the poststructuralist movement, rejects both of these approaches and accepts the “subject” understanding as the fundamental point of power. In this context, it is revealed that power is a micro-structured concept and exists at every level of society. Power exists in different forms in all social relations, and it penetrates into the finest details of the subjects’ lives through the bodies of the subjects by wrapping the daily life like a web (Agamben, 2017). According to Foucault (2014) power does not only work through legal, representative, physical or economic power, but also with the impact of norms, science and political technologies; it is a plural, relational and productive mechanism where body and soul functions together. Here, rather than a structure where dominance is unilateral, strategy applications is in play based on competing and resisting various parties aiming at establishment of a balance of power. In other words, instead of nurturing bodies with violence and death, power chooses to make them healthier and more effective and evolved into building a society which is in better compliance with norms.

A phenomenon of power is at the focal point of the discursive or non-discursive practices of the acts that people experience as subjects. However, contrary to traditional discourse, Foucault does not attribute a negative meaning to the concept of power. In addition to this, he emphasizes that the modern state has transformed from a land-based structure to a population-based one in recent years, while the biological and health life of the people gains importance. Thus, managing people becomes easier (Agamben, 2017). The bio-power structure, which emerged simultaneously with the establishment of nation states, expresses the function of regulating the methods to be applied for the rehabilitation of bodies, immigration problems, public health, housing facilities, family and population under control in constantly changing and developing societies, with some statistical formulas. While controlling individual on one hand, bio-power strengthens them on the other. Foucault (2007) not only sees bio-power as intervention in the private or social life; on the contrary, he thinks that it consists of steps taken to create societies anew which adopt and internalize bio-power. In order to maintain the continuity and functioning of the power, surveillance and control over the subject is required without moving away from the axis of knowledge and power. There is a strong relationship of reciprocity between seeing and power. Foucault approached this relationship with the question “Is she who sees is in power, whether she is seen or seen without being seen?.” Thus, he distinguished the basis of being in power as seeing without being seen. He explains this mechanism with the analogy of Panopticon inspired by architecture. This concept, which is formed by the combination of the words “Pan” and “Opticon” and means “to observe the whole,” expresses the transformation of the eye into the power space and points to the existence of an important control system. Panopticon, considered as the control symbol of the digital age, transforms the routine behavior of individuals in daily life into data (Gehring, 2017). Also, panopticon design represents a key concept in explaining the emergence of self-disciplined and contemporary societies and governments. According to Foucault (1995), who both realized the modern transformation and tried to express the power that holds control, the panopticon, which is the fundamental basis of liberal government, is the mirror of modernity. The most important aspect of panopticon, which includes several functions such as isolation, imprisonment, detention, forced labor or education, is its authoritarianism. Hospitals, jails, schools and similar structures are examples of panopticon prisons. As the prison analogy evokes, the individual who feels bio-power by thinking that her every step is controlled and monitored, begins to develop her self-control. Subjects who think that they are constantly being monitored and controlled try to bring normality to their behavior, thus internalizing the power. Therefore, there is no need for an external intervention tool for individuals who have achieved self-control. Power defines the notions of normal and abnormal and places normal ones in a privileged position over others.

Management has gained legitimacy as the managed gave the right to manage with their consent. Humans are thinking beings and it is natural that they sometimes show resistance to power. As a result, power is in constant transformation depending on changing conditions and the resistance it receives. According to Foucault power is a process of normalization without resorting to force. The instruments used in this process are hospitals, prisons, army, education institutions and the legal system (Galal, 2017). These institutions allow for the raising of cadres required for normalization process. These institutions are used in order to raise the subjects required for “normalization” process by distinguishing the normal ones from others. It places those who act against rules of law into prisons, the mentally ill into mental facilities, and teaches all rules of the power to the new generations; so, it tries to complete the normalization process. By categorizing the subjects into normal (submissive to power) and abnormal (non-submissive to power), it strengthens the legitimacy of the power by showing that the abnormal are pushed out of the system through prisons and hospitals (Çelebi, 2013).

Foucault says that power performs legitimation through discourse. There is a mutual relationship between discourse (the language used in spoken and written expressions) and social structure and institutions which is based on shaping each other. In addition, the role of discourse is to serve the maintenance of status quo (Ladkin & Probert, 2021). Through discourse, unequal power relations between gender, social class, women, men, economy, racism, cultural difference, ethnic minority and the majority are produced (Fairclough & Wodak, 1997). Thus, discourse has an ideological character. Power, holding information in its hands, creates its own discourse and the discourse of power is produced through all institutions. However, this production does not remain the same, power produces new ones in accordance with the resistance it meets; thus, the course in which the struggle and power balances are tried to be established is followed.

Until the leader mobilizes her motivation power in individuals for certain purposes, management activities are in the form of a lifeless cocoon. The characteristics of a good leader can be divided into two as external and internal. External characteristics of the leader are seriousness, learning ability, compromise, setting the agenda, trust, kindness and simplicity. His/her inner characteristics are self-knowledge and peace, being open to criticism, and resilience (as cited in Colcaud 1999, Arklan, 2010). Similarly, gender factor is also important in creating leadership style. Although men are identified with the word “leader” due to stereotypes in traditional societies, today the number of women in executive positions is increasing. With this increase, research has revealed how the gender variable reflects on the style of leadership (Lemoine et al., 2016; Martinez-Leon et al., 2020; Mroz et al., 2018; Topic et al., 2017).

It is claimed that crisis environments are the periods when leaders are most seriously tested (Klann, 2003; Schoenberg, 2004). A good leader must be able to put into effect emergency and crisis management plans in the shortest time possible so as to decrease the effect and duration of a crisis. Some crises can be predicted, but not every crisis is predictable. The way the leader interprets problems and his/her attitude to solving problems can determine both the success in solving problems and the model of behavior in which society will unite in the face of this problem. This situation becomes more important, especially during crisis periods (Gezgüç & Duman, 2020). There are different types of crisis categories (political, organizational, social, technical, natural) in the literature depending on the causes and consequences of crises. However, there are also rare types such as sudden, silent and permanent crisis, perceptible and strange crisis, and triggering crisis. Leader’s attitude in crisis situations is not monotonous. The style of leadership may also change depending on the course of the process, just like a master who takes out the necessary tools from his bag (Asuncion et al., 2006). Leaders can influence individual beliefs and behaviors through different channels: by reducing information asymmetries and thus minimizing coordination problems, establishing a social norm or simply message through emotional and symbolic transmission messages (Ajzenman et al., 2020). It is expected that in times of crisis from leaders, it will be able to quick and effective decisions, improve interagency dialog and coordination, prepare for possible scenarios, and help to accurately inform and direct the masses (Gezgüç & Duman, 2020).

Research on leadership styles has shown that women are more collaborative, intuitive, sensitive and empathetic than men (Peterson & Bartels, 2017). Female caretakers and “caregivers” for health may be more effective and humanitarian than male, risk-taking “warriors” in the fight against the invisible viral enemy that can bring nations to their knees (Luoto et al., 2021). Sergent and Stajkovic (2020) found that states with women governors had fewer COVID-19 deaths than states with male governors and that states with women governors responded better when governors ordered early home leave, as evidenced by fewer COVID-19 deaths. According to the study, female governors were more empathetic and confident in their briefings, which may be a possible mechanism for this effect. Bruce et al. (2022) adds to the body of evidence pointing to positive effects of female leadership on policy outcomes in a variety of domains. In particular, women leaders were shown to outperform men during a major crisis. COVID-19 outcomes are systematically and significantly better in countries governed by women, according to Garikipati and Kambhampati (2020), which may be explained in part by the proactive policies they have adopted. Even taking into account the institutional context and other constraints, women’s leadership has given countries an advantage in the current crisis. Aldrich and Lotito (2020) pointed out that in countries where women are more actively involved in the political decision-making process, different political outcomes may be achieved, possibly reflecting a higher level of gender equality in society. As a result, governments in which more women are represented at all levels may be more aware of the social and economic impacts of school closures, such as the potential impairment of children’s development or the gendered impact of the loss of child care.

During the COVID-19 pandemic, which is one of the biggest pandemics of the last century and can be regarded as a global tragedy, leaders had to mobilize many resources of their countries and persuade their citizens in all social areas to change their routine behaviors (Huber & Helm, 2020). The approach the country leaders adopted in their efforts to persuade their people has been an important factor in their success. In this sense, some leaders have behaved in a more agile, courageous and determined manner while dealing with the pandemic. The communication of the said leaders with the media and the public is considered as calm, affectionate, honest, empathetic, science-oriented and transparent. Political speeches by women leaders around the world in mid-March 2020 helped shape the global perception that women leaders control the spread of the virus better than men. These leaders have used familiar, prosocial, feminine frameworks in their discourses that are oriented toward the double political bond and resonate with the citizenry. The speeches of the female leaders were also more coherent and had more social and community themes than those of the male leaders (Windsor et al., 2020). During the COVID-19 pandemic, it is seen that the number of female leaders among the leaders who are successful with their leadership attitudes and behaviors both in the country administration and in local governments is higher (Aldrich & Lotito, 2020; Dada et al., 2021; Garikipati & Kambhampati, 2020; Huang, 2020; Meagher et al., 2020). Within the scope of the research, it is aimed to determine how female leaders control bodies with their discourses and behaviors on the basis of their leadership qualities.

## Method

The method of this study is a discourse analysis, which is a qualitative research pattern, aiming at researching power, hegemony, ideology, force and similar topics in the context of text and verbs and to reveal the underlying implicit messages. It is an approach that analyzes the ways and functions of using action patterns in the language, rather than analyzing individual words or sentences, and therefore evaluates the discourse as a whole (van Dijk, 1983). The main approaches of discourse analysis are shaped on the basis of critical school, positivism and phenomenological perspective, and interpretative, descriptive strategies, subjectivity, uniqueness, and autonomy (Taylan, 2011). The phenomenological approach was taken as the basis of the study, which examines the discourses and leadership styles of four female leaders selected from among the countries that have a female president in the face of the COVID-19 global pandemic. The source of the documents used in this review is within the scope of the secondary documents, as they are newspaper articles, scientific articles and official websites. So, the total number is 13 news reports published from January to October 2020. The analysis is divided into three stages to obtain an in-depth analysis. Firstly, news reports headlines were examined as they are the first elements that gain the readers’ attention. Then, the full-text stories of news were evaluated to understand the whole story. The last stage is to reveal the leadership styles of women leaders manifested in the discourses of politics. With these documents, the opportunity of controlling the individual given to the governments by the pandemic was evaluated through Foucault’s concept of panopticon. Therefore, through the discourses of female leaders who have been successful in combating the pandemic, behavioral patterns were tried to be revealed on the basis of leadership characteristics. The statements of the female leaders of the countries studied (Finland, Iceland, Taiwan and New Zealand) were conveyed in a holistic manner and general interpretations have been made.

## Findings and Discussion

A detailed analysis was made on the interpretation of many points obtained in the study and what they mean in a planned manner.

### Finland

Finnish health officials reported the first case in a Chinese woman aged 32 who came to the country from Wuhan on January 24, 2020. Number of cases slowly increasing, the country declared a state of emergency as a response to the pandemic. On Wednesday April 29, 2020, Prime Minister Sanna Marin made a statement to the parliament including detailed information on handling the COVID-19 crisis and said that they took extraordinarily wide-range and serious restrictive measures, the purpose of which was to prevent the spread of the disease among the population, to not overload the health system of the country under any circumstances, and especially to protect the lives and health of people under risk with underlying conditions. In her speech, Marin stated: “*Our aim is still to prevent the spread of the virus in Finland, to preserve the capacity of the health system and to protect people, especially those most at risk. Efforts to prevent the progression of the pandemic in Finland have so far been successful. However, not all restrictive measures can be lifted once, because the situation is still serious*.” In addition, she added that they succeeded in slowing the spread of the pandemic and adapting their actions to the changing situation.

As a result, the government believed that Finland could move gradually and in a controlled manner from a situation in which there are comprehensive restrictive measures in society to a situation where outbreak management is raised to an advanced level under the “Contagious Diseases Act.” Although Finland is a country with a high level of preparedness for different situations compared to most other countries, Marin admitted during her speech that they did not predict that the crisis would be so deep and serious at the beginning of the year. The fact that they have increased the level of preparedness in health services and the carrying capacity of intensive care can be an example to this situation. She continued her speech as follows: “*In Finland, although the situation is moderate thanks to restrictive measures, it is still not the time to take a deep breath. The situation is still very serious. The disease is unpredictable and there are many things that we do not know about this coronavirus. Many Finns wonder when restrictive measures will be lifted so that we can return to normal life. This is a very understandable question and I wish I could give a clean and clear answer. But the truth is that we still do not know how long the situation will take and when we will be able to return to our normal lives. Although we managed to suppress the disease in Finland, it can come back later. In one way or another, we have to learn how to live with the virus for now.”* As such, she warned the people to not let their guard down (Government Communications Department, 2020).

The hybrid strategy of Finland in her struggle with the pandemic focuses on the lifting of restrictive measures in a controlled manner as well as a “*test, trace, isolate and treat*” approach. As a part of this hybrid strategy, the government continues preparations for presenting a mobile application which will be used in the management of the pandemic. The preliminary condition is that it is voluntary and the privacy can be protected (European Centre for Disease Prevention and Control, 2020).

Marin says the government made a mistake with the Helsinki-Vantaa airport in the midst of the crisis: “*The uncertain situation that has been going on for too long should have been resolved more quickly. When mistakes are made, we must examine them and learn from them. (…)We still face challenges in obtaining protective equipment and healthcare supplies, both in Finland and in many other countries, but we are looking into solutions to deal with this issue. It is absolutely certain that mistakes will also be made in the future. This is because we cannot know everything or predict all possible scenarios. People also fail and make mistakes because we are human. It is "extremely important" that we record our experiences in this crisis. We must learn lessons from the crisis and, once the acute situation is over, we must carefully assess the functioning of our central government, cooperation between authorities and our national preparedness for emergencies. Based on our experiences so far, I can say that we have lacked the instruments needed to deal with times of crisis, and we have had to create them along the way under far too tight a preparation schedule. Our emergency powers legislation must be carefully assessed and reformed in cooperation with all parliamentary parties. The legislation as a whole should be revised from the point of view of preparedness so that we can be better prepared for possible crises in the future.”* With this speech, she expressed that they made mistakes and that they had to learn from these mistakes. Before finishing her speech, Marin did not neglect to thank the public: “*We would not have succeeded in curbing the spread of the pandemic in Finland without the citizens’ commitment to complying with the restrictive measures. People have acted responsibly and have responded to the situation with the seriousness required by it. For this, I would like to thank each and every one of you!”* She also showed her gratitude with the following words: “*It is important to make sure that the burden caused by the crisis is shared in a socially, economically and environmentally sustainable way, and that it is divided fairly between the generations. I would also like to thank the numerous healthcare workers, teachers, cleaners, police officers, public transport drivers, retail and catering workers and many other groups of professionals and Finnish businesses that are responsible for keeping society functional in the midst of the crisis”* (Government Communications Department, 2020).

Sanna Marin holds the titles of the world’s youngest prime minister and the country’s third female prime minister. The pandemic broke out in a very short time after she was elected as the 46th Prime Minister of the country on December 10, 2019. Marin declared a state of emergency as the number of cases started to increase. Then, after receiving the necessary information and recommendations from field experts, she updated the country’s strategy to combat the pandemic. The strategy was designed on three themes: to not expose the health system of the country in trouble, to prevent the spread of the pandemic, and to protect people and especially those at risk. Although Finland is a country with a social security system, strong economy and policies that it has formulated for different situations, Marin’s admitting that they could not predict that the crisis could be so big and deep reflects Marin’s characteristic of being an honest leader. Empathy is very important for successful leadership, especially in uncertain times (Huang, 2020). In the content of her speeches to her people who were psychologically affected by the situation, she emphasized that they should learn to live with the virus by approaching them with empathy. She demonstrated her persuasion skill by speaking effectively about being patient and enduring difficulties. The fact that she did not make using developed technological applications compulsory is an important example of her sensitivity to moral and human values. Despite the fact that leaders are expected to be visionary, to make projections for the future, and to see threats, Marin honestly stated that not everything can be predicted, possible scenarios cannot be produced for every situation, and that it is natural for leaders to make mistakes because they are human beings. However, by drawing lessons from all the foregoing, she stated that it is necessary to be prepared for possible future crises in cooperation with all relevant institutions, within the framework of sharing duties and devolution of authority. She also stated that instead of taking measures after the crisis breaks out, which is the case in Covid-19 crisis, it is necessary to establish a standard for coping with crises in agreement with all parties in the Parliament and to analyze and rearrange existing legislation. As the pandemic was accepted by all layers of the people who fulfilled what the leader and government asked for, Marin thanked her people without discriminating anyone.

### Iceland

Iceland can be shown as an example with the strategy she followed during her fight against C-19. They acted immediately after it was heard that the virus emerged in the world, made their preparations, and started to wait for the identification of the situation. The first case in the country was seen on February 28, but individuals had begun to have their tests made, which prevented the anxiety, panic and uncertainty other countries suffered. It was only a waiting period to see when the first case would occur. Then, the second stage in the fight against the pandemic would be assumed. With the pandemic making itself felt, measures were taken in the country. On March 24, swimming pools, bars, fitness centers and museums were closed and more than 20 people have been banned from gathering. After the first case, the goal was to end the pandemic at the end of April.

Iceland reached this goal with the note “*no cases have been detected in the last 24 hours*” on April 23. In her speech on FRANCE 24 channel, Katrín Jakobsdóttir said: “*The pandemic is not over in our country yet, but I think we have taken control of the pandemic with very few new infections*.” Jakobsdóttir explained the reasons why her country achieved this as collective tests, compliance of the population with social distance rules, and contact tracing (Perelman, 2020). In Iceland, which has a population of 364 thousand, it was much easier to apply random tests to individuals compared to countries with a large population. A second importance of these tests is that Icelandic people do not show any of the symptoms of the disease. A 2 m limit has been imposed to maintain social distance. Iceland, which took measures such as reducing the class size and shortening the course hours, did not close educational institutions other than universities. In addition, Jakobsdóttir stated that they carried out contact tracing through the smartphone application and achieved 93% success. She added that the privacy of individuals is not harmed because the public accepted this practice on a voluntary basis (Perelman, 2020).

Jakobsdóttir emphasized that the key to dealing with the coronavirus for a politician is to approach the crisis by getting rid of their egos. In this context, Jakobsdóttir said the following in the “TIME 100 Talks” interview with journalist Katie Couric on May 21: “*What we can learn from this is that as a politician it’s important to put your ego aside, and learn from humble scientists who are faced with a crisis that no one can expect.*” Taking the rise of the tendency to listen to the voice of science as an opportunity in this process, Jakobsdóttir expressed that she hoped this would reflect on other global problems such as the climate crisis: *“The priority of the climate issue has never been more important. We can use some of the lessons learned in this pandemic in the fight against the climate crisis.”* Jakobsdóttir pointed out that despite being a small country, the society showed a great example of solidarity by cooperating in combating the pandemic, and thanked her people for fully complying with the measures and recommendations taken by the government and added: “*You could say that the responsibility is placed on each of us. We all have to be a part of it if it works, and I think it really worked.*” (Haynes, 2020). Jakobsdóttir, who became the prime minister of Iceland on 30 November 2017, was also elected President of the Council of Women World Leaders on 20 February 2020.

Prime Minister Jakobsdottir, who has been shown as an example to other countries with her successful fight against COVID-19, immediately mobilized her country by using her foresight against possible threats and risks that await in the future despite the uncertainty of the sudden outbreak. After the emergence of the virus was heard, she started testing her citizens without waiting for a case to be detected in the country. Strong and transparent leadership helps reduce citizen anxiety in times of crisis. Therefore, the environment of chaos, anxiety and fear seen widely in other countries has disappeared to some extent. After the detection of the first case, Jakobsdóttir made some restrictions in the country and evaluated and analyzed the issue of stopping the pandemic within the framework of the characteristics of her country, and explained her ambitious goal for the future to citizens. Thinking positively that the contagion would end in the country, she targeted April and warned her people not to let go of the measures even though on April 23 this target was reached, which she showed after the first case on February 28. She also used technology in Iceland to produce some solutions during the pandemic period.

In the face of this pandemic that affects the whole world, she believed in the importance of a leader to abandon her egos, follow the recommendations of scientists, and be transparent in decisions. She persuaded people to continue this struggle in a team spirit by ensuring that this belief was reflected in her effective speeches to her people. At the same time, she considered turning the COVID-19 crisis into an opportunity, and shared the idea that the conscious struggle for the pandemic in a team spirit could also be applied to global climate change.

### Taiwan

Taiwan is a country populated by some 23 million people which is geographically close to the city of Wuhan of China that is considered as the origin of the pandemic. The country is 17th in the world in terms of population density with 649 people per square kilometers. In Taiwan, which is located on a mountainous island, the population density in the cities is even higher as cities can only be established on the coasts (Capital Taipei9,918 people/km^2^). In addition, while the size of the pandemic is expected to be larger, as the Asian Continent is one of the important technologies and tourism destinations, it has been one of the countries least affected by the virus. In particular, voters coming from abroad to vote in the Taiwan Presidential elections held on January 11, 2020 forced the government to take measures against possible infectious diseases. In this context, measures were taken against passengers and crew coming from Wuhan since December 31st. The government launched the Central Epidemic Command Center (CECC) on January 20 to coordinate inter-agency cooperation, and the first case was detected on January 21.

After the first patient was diagnosed, the government raised the security level and recommended not to travel to Hubei Province. In the first days of February, a mask supply problem emerged in the country (Farr & Gao, 2020). Software engineer Howard Wu noticed in the messages on her social media account that people’s stress levels increased due to C-19. In LINE, one of the country’s popular messaging apps, people were asking which stores have masks stock. Inferring from the messages of unknown origin from the messaging application, he prepared a web page using the Google Map application, showing the stores with mask stock in “green” and the stores without mask stock in “red.” About 1 month before the World Health Organization declared a global pandemic, the use of this application started to spread among the Taiwanese people. While the news of the deaths of heaps of people was coming from Wuhan every day, Taiwan was on the red alert and Wu’s mask map became popular day by day. Minister of Digital (Audrey Tang), an open-minded person who believes in technology and is aware of these practices, ensured that the National Health Insurance system was integrated into this application. Therefore, thanks to the “mask vending machine” installed in almost all major cities of the country, those with national health insurance started to buy their 2-week masks without waiting in line in pharmacies. Minister Tang stated that this was a project activated by collective consciousness (Leonard, 2020).

At the same time, distributions were shared and production accelerated to prevent panic buying of face masks. In February, the government partnered with the Machine Tool & Accessory Builders’ Association and manufacturers to invest in new machines to produce surgical face masks. Manufacturers were obliged to resell the masks to the government at an agreed fixed price. Thanks to this effective government-led collaboration strategy, Taiwan is currently able to produce around 20 million masks a day. Taiwan’s National Health Insurance Administration and the National Immigration Agency worked together to identify suspicious cases, recording the medical and travel histories of individuals. As of the end of March, everyone new to the country was subjected to a 14-day quarantine rule. CECC also monitored individuals with a smartphone app, and in addition to the 24-hr hotline, it created a chat bot by collaborating with two technology companies (HTC and LINE) to inform people about their health and provide advice about the virus. Local authorities were responsible for asking questions and, if necessary, bringing essential daily supplies to the quarantined citizens to learn about their health. In addition, police organization undertook the task of quarantine and controlling the implementation of the rules (Hsieh & Child, 2020).

In April, Taiwan President Tsai Ing-wen said the following in her statement as part of the “TIME 100 Talks” coronavirus talks: *“Taiwan is an island of resilience. Centuries of hardship have compelled our society to cope, adapt, and survive trying circumstances. We have found ways to persevere through difficult times together as a nation, and the COVID-19 pandemic is no different. Despite the virus’s highly infectious nature and our proximity to its source, we have prevented a major outbreak. As of April 14, we have had fewer than 400 confirmed cases.”* Tsai stated that the country, which has not been a member of the World Health Organization since 1972 for political reasons, is still willing to share its experiences with other countries: *“Indeed, Taiwan has effectively managed the containment of the corona-virus within our borders. Yet on a global level, COVID-19 is a humanitarian disaster that requires the joint efforts of all countries. Although Taiwan has been unfairly excluded from the WHO and the U.N., we remain willing and able to utilize our strengths across manufacturing, medicine and technology to work with the world.”* In addition, she finished her words as follows: *“Global crises test the fabric of the inter-national community, stretching us at the seams and threatening to tear us apart. Now more than ever, every link in this global network must be accounted for. We must set aside our differences and work together for the benefit of humankind. The fight against COVID-19 will require the collective efforts of people around the world”* (Tsai, 2020).

Since the outbreak of the pandemic, different practices have been tried by the Minister of Health, Chen Shih-Chung, at the press information conferences which have been organized on a daily basis. For example, the press conference on May 15, 2020 was held as “specific to children” and 11 selected elementary school children asked direct questions to the Minister of Health. In the first days of May, the “contactless treatment” technology was developed by Taipei Medical University Hospital and the Industrial Technology Research Institute aiming at protecting healthcare workers from the virus and started to be tested. Within the scope of this application, which allows patients to be monitored with artificial intelligence, cameras and infrared sensors, it is aimed to minimize the contact of healthcare professionals who have to check on the patient repeatedly during the day with the new technology.

The government earned high level of confidence of the public with the measures it developed in coronavirus crisis. According to YouGov survey conducted in Taiwan in May, it was revealed that the people’s trust in the government and health professionals as regards C-19 was over 80% (Hsieh & Child, 2020). In addition, since the first day of the pandemic, paramedics contacted the public regularly and gave daily briefings, and started to have these briefings weekly in June (Farr & Gao, 2020).

Since the outbreak of the pandemic, declaration of a curfew by Central Pandemic Command Center has been evaluated as a very low possibility, and Taiwan displays a highly successful appearance with 937 active cases and nine loss of lives as of February 12, 2020 (Worldometer, 2021). Taiwan, having lost 73 people in the 2003 SARS pandemic, has based its fight against Covid-19 on this experience.

Tsai, who received the title of the first woman and single President of the country when she was elected for the first time in 2016, was reelected on January 11, 2020, and the outbreak of C-19 pandemic followed immediately. Taking a very short time, Tsai shared the responsibility by collaborating with the necessary institutions in order to control the spread of the pandemic. After the first patient was diagnosed, the excessive demand for masks and disinfectant products was placed under the control of the government. In addition, the mask map application was launched after people asked each other through their social media accounts about the stores they could find masks. The president, who attaches importance to entrepreneurship, did not ignore the efforts of a citizen to develop this application and supported him. Thus, the problem of mask supply was overcome, thanks to the vending machines placed in the major cities of the country. Since Taiwan is represented by China in the United Nations, it does not have access to the World Health Organization’s database. Although access to this database was provided by the Chinese government for a while, this access was cut off because the Tsai government opposed the “One China” policy in 2016 when it took office. Its inability to work with WHO has kept Taiwan in a state of preparedness for any health crisis that might arise since the SARS Crisis in 2003. Tsai’s support to utilize all possibilities of technology at the highest level in this process has been an added value for the country.

### New Zealand

The prime minister, Jacinda Ardern, declared that the first COVID-19 case was detected in a New Zealand citizen who returned from Iran on February 28, and initiated an unexpected quarantine process by saying “*we can stop the spread by staying at home and reducing contact*.” Ardern called the entire country to come together to protect their families, friends and neighbors, calling its public “a team of 5 million people.” The reaction of the country to C-19 is one of the most effective and rapid measures taken among the countries of the world. Health Minister Dr. Ashley Bloomfield stated in a statement that the country’s C-19 strategy consisted of rapid tests, contact analysis and isolation, and strict compliance with the rules in the public health guide. After the World Health Organization declared the coronavirus pandemic “International Public Health Emergency” on January 30, the government began implementing outbreak prevention measures, and continued to strengthen them in the following weeks. Although they had a pandemic plan prepared for flu pandemic, they decided that they would not be able to overcome C-19 pandemic applying this plan and took rapid steps. For this reason, the government adopted a cautious approach and on March 26 asked the people except health professionals and compulsory service providers to impose self-quarantine at their homes (World Health Organization, 2020). In addition, media organs constantly informed the people about C-19 and told them what they had to do.

As of April 9, the number of people who overcame C-19 began to surpass those who contracted C-19 on a daily basis, and May 21 became the first day when no cases were witnessed. With the awareness of giving consistent and clear information to the public on the pandemic, Andern delivered a speech to her people in June and said “*Today I’m speaking directly to all New Zealanders to give you as much precision and clarity as we can when dealing with Covid-19*.” Praising their struggle against C-19 and saying that she made a “little dance” when she learned that all the patients in her country had recovered, Ardern said, “*This is not over, but we cannot deny that this is a great success. I end my word very simply. Thank you, New Zealand*” (Hislop, 2020).

Ministry of Health and Ministry of Foreign Affair and Trade worked together with Australia and World Health Organization to protect the island countries in Pacific which are not affected by C-19 pandemic and provided material and distance education support to the health professionals there.

The prime minister announced at the Malaghan Medical Research Institute in Wellington on August 27 that the country would invest hundreds of millions of dollars to ensure access to individuals as soon as a vaccine is available. Ardern concluded as follows: “*We’re working to make sure we have the capacity to produce a vaccine on a large scale in New Zealand too. We work particularly closely with Australia in all phases of vaccine development, distribution and use, and we help our Pacific neighbors to make their voices heard. Vaccine nationalism only helps the virus*’’ (Pasley, 2020). The parliamentary elections to be held in the country due to the coronavirus pandemic were postponed to 17 October. She apologized to her public for the maskless selfie she took with construction workers and without complying with the social distance rule in Aucland, where she visited on September 21 as part of her election campaign: *“I paid a lot of effort not to shake hands with people. I am trying too hard to protect social distance. In that particular photo, I made a mistake. Yes, I should have moved further forwards and I should have asked them to step apart as well.”* (Workman, 2020).

Jacinda Ardern, elected as New Zealand’s third female prime minister on October 26, 2017, is the world’s second prime minister who gave birth during her term of office. Ardern first appeared on the world agenda with her attitude toward the attack on two separate mosques in Christchurch on 15 March 2019. She described the biggest attack in the country’s recent history as a terrorist attack, and despite being an agnostic, she portrayed an influential world leader who exemplifies solidarity with the phrase “We are all one, they are us.” Ardern first announced the pandemic to her people and stated that they would work together as a “team of five million” in order to protect the health of everyone, thus united her people in a collective good effort. She thought that the current pandemic plan would be insufficient in such a pandemic, and determined her strategies, being aware that the future would bring economic and social costs. Ardern successfully implemented all aspects of effective leadership communication. She displayed her joy by dancing at the moment she explained that no case was seen, which shows that she was portraying a sincere, dynamic and intimate leader with her people. Ardern has succeeded in proving that the leadership model that believes that leaders should be tough and that hardness eliminates courtesy is wrong within the framework of her behaviors. General public support for the New Zealand government’s approach has so far been spectacularly high, especially since Ardern receives generally positive press coverage internationally, but his government’s popularity domestically has never been particularly high: It was not until mid-February that an influential poll showed that while Ardern had the incumbent’s “prime minister” advantage at the time and received 42% support, the two main opposition parties combined would have enough support to govern if the results of the survey translate into a real choice. However, this rate remained at only 59% in the G7 countries (Wilson, 2020).

## Conclusion

The countries studied in this paper are different from each other in terms of economic, social and military opportunities. At this point, the countries examined have been considered in terms of their success in combating the epidemic by accepting the existence of the epidemic in a short time and using their resources most effectively. COVID-19 pandemic provided an environment where the degree of ability of world leaders to develop strategy against several interrelated, intertwined challenges could be measured. Some of the leaders stumbled whereas others elevated their success to higher levels. A general analysis of 194 countries shows that countries with female leaders experienced lower cases and death rates compared to other countries. In other words, countries led by women have also been more successful in combating COVID-19. This popular situation is also often discussed and confirmed by different scientists in the world’s leading sources; for example, New York Times, the Harvard Business Review, Vox, Forbes, Stanford Medicine, and NBC News (Windsor et al., 2020). In addition to this, according to the Global Gender Equality Report (2020), countries with high gender parity (where women participate in society, employment, receive higher education) are also successful in the Covid-19 pandemic, and that the success of countries led by female leaders in this context may be due to this social structure (Croteau & Champoux-Paillé, 2020). It has been determined that female leaders have some aspects in common in terms of this achievement. During the pandemic, leaders had to rapidly mobilize several resources of their countries and persuade individuals in changing their habits and routines. Leaders used effective and persuading means of communication so as to ensure engagement of the public and their voluntary compliance. In their leadership and leadership communications, women presidents exemplified truth, openness, determination, social media, technology awareness, empathy, and thoughtfulness. They involved everyone in their discourse and behavior to deal with this crisis in cooperation, regardless of language, religion, race or age. Strong reliance on facts has brought rational optimism with it. Leaders have built up public confidence in themselves, as they accurately conveyed the information they received from experts on infection to their citizens day by day (Huang, 2020). Therefore, it was observed that they obeyed the rules and warnings more meticulously. When the data obtained from the epidemiological modeling of field experts and the measures taken by the leaders combined, the countries had lower contagion speed and mortality rates. It is both difficult and very important to act decisively in times of crisis and uncertainty. However, this does not mean that the leader will act alone; road maps created with the suggestions of the relevant people in difficult times gain importance. It is seen that female leaders have achieved success in this area as well. As the process continued, leaders also benefited from the solutions produced by technology. Social media accounts encouraged those who prepared artificial intelligence and smart phone applications and pioneered their use. The concept of the bio-power, which began with the emergence of nation-states, the observation, tracing, and control of the subjects by the rulers have been carried to an advanced level with the use of this technology. Therefore, the question remains whether the same control will continue when the pandemic is over. In addition, it has been revealed that leaders differ by gender in communicating with the public on issues related to C-19. The phrase “fight,” which is commonly used in speeches about the pandemic, is a masculine metaphor and mentality. Fighting is a concept related to an enemy, and countries have now declared “war against an invisible enemy.” However, female leaders did not include this phrase in their statements, but emphasized that they would overcome the crisis by struggling together in solidarity. As a result, in today’s world when the populist and autocratic leader type is on the rise, female leaders have succeeded in inspiring other countries as well as making their countries successful. More importantly, the struggle of female leaders against the pandemic has revealed that another form of management is possible.
